# Primary exposure to SARS-CoV-2 protects against reinfection in rhesus macaques

**DOI:** 10.1126/science.abc5343

**Published:** 2020-07-02

**Authors:** Wei Deng, Linlin Bao, Jiangning Liu, Chong Xiao, Jiayi Liu, Jing Xue, Qi Lv, Feifei Qi, Hong Gao, Pin Yu, Yanfeng Xu, Yajin Qu, Fengdi Li, Zhiguang Xiang, Haisheng Yu, Shuran Gong, Mingya Liu, Guanpeng Wang, Shunyi Wang, Zhiqi Song, Ying Liu, Wenjie Zhao, Yunlin Han, Linna Zhao, Xing Liu, Qiang Wei, Chuan Qin

**Affiliations:** 1 Beijing Key Laboratory for Animal Models of Emerging and Remerging Infectious Diseases, NHC Key Laboratory of Human Disease Comparative Medicine, Institute of Laboratory Animal Science, Chinese Academy of Medical Sciences and Comparative Medicine Center, Peking Union Medical College, Beijing, China.; 2 Department of Radiology, Beijing Anzhen Hospital, Capital Medical University, Beijing, China.

## Abstract

Coronavirus disease 2019 (COVID-19), which is caused by infection with the severe acute respiratory syndrome coronavirus 2 (SARS-CoV-2), has become a global pandemic. It currently remains unclear whether convalescing patients have a risk of reinfection. We generated a rhesus macaque model of SARS-CoV-2 infection that was characterized by interstitial pneumonia and systemic viral dissemination mainly in the respiratory and gastrointestinal tracts. Rhesus macaques reinfected with the identical SARS-CoV-2 strain during the early recovery phase of the initial SARS-CoV-2 infection did not show detectable viral dissemination, clinical manifestations of viral disease, or histopathological changes. Comparing the humoral and cellular immunity between primary infection and rechallenge revealed notably enhanced neutralizing antibody and immune responses. Our results suggest that primary SARS-CoV-2 exposure protects against subsequent reinfection in rhesus macaques.

Coronavirus disease 2019 (COVID-19), which is caused by the severe acute respiratory syndrome coronavirus 2 (SARS-CoV-2), emerged in China and spread throughout the world causing a global pandemic (*1*, *2*). Some patients who were discharged with undetectable SARS-CoV-2 have reportedly had a positive result upon subsequent tests (*3*–*5*). Recently, SARS-CoV-2-specific neutralizing antibodies (NAbs) were detected around 10–15 days after the onset of COVID-19 (*6*–*8*). The possibility that patients have a risk of “relapse” or “reinfection” after recovery from the initial infection has raised concern. In this study, we therefore used nonhuman primates to longitudinally track the short-term infectious status from primary SARS-CoV-2 infection to reinfection by the same viral strain.

Seven adult Chinese-origin rhesus macaques (M0 to M6, 3–5 kg, 3–5 years of age) were modeled for challenge–rechallenge observations. Six monkeys (M1 to M6) were intratracheally challenged with SARS-CoV-2 at 1 × 10^6^ 50% tissue-culture infectious doses (TCID_50_). After undergoing a mild-to-moderate course of SARS-CoV-2 infection, and transitioning into the recovery stage from the primary infection, four monkeys (M3 to M6) were rechallenged intratracheally with the same dose of the SARS-CoV-2 strain at 28 days post-initial challenge (dpi). The remaining two monkeys (M1 and M2) with primary infection were not rechallenged and were used as the negative control of the rechallenge group. A healthy monkey (M0) was given an initial challenge as a model control of the second challenge. The pathological changes with viral-dependent distribution were compared using necropsy specimens between two monkeys that underwent only the initial challenge (M0 at 5 dpi and M1 at 7 dpi) and two monkeys that underwent challenge–rechallenge (M3 and M5) at 5 days post rechallenge (dpr, 33 dpi). Body weight, rectal temperature, nasal/throat/anal swabs, hematological measurement, chest X-ray, virus distribution, pathological changes, and the analysis of immunocytes, and binding and neutralizing antibodies were examined at the designated time points (Fig. 1). Weight loss ranging from 200 to 400 g was found in four monkeys that underwent the initial challenge (4/7, M0, M1, M2, and M4) (Fig. 2A), whereas the rectal temperature was not elevated in any of the monkeys (0/7) (Fig. 2B). Reduced appetite and/or increased respiration were common (6/7, the exception of M4), but emerged transiently and exhibited a very short duration. Regarding viral dissemination, the peak viral load (6.5 log_10_ RNA copies/mL) in nasal swabs and pharyngeal swabs was detected at 3 dpi, followed by a gradual decline (Fig. 2, C and D). The peak viral load (5 log_10_ RNA copies/mL) in anal swabs was observed at 3 dpi, followed by a linear decline to reach undetectable levels at 14 dpi (Fig. 2E). In all monkeys that received the initial challenge, white blood cell count (WBC, 3.5–9.5 × 10^9^/L), lymphocyte counts (LYMP, 1.1–3.4 × 10^9^/L), and neutrophil counts (NEUT, 1.8–6.4 × 10^9^/L) fluctuated within normal ranges. Compared with the baseline, a slight but significant reduction in WBC and LYMP was observed after the primary infection (Fig. 2F). On radiological examination, bilateral obscured diaphragmatic surface and decreased transparency of lung fields with a small patch shadow in the left lower lobe were detected, indicating mild-to-moderate interstitial infiltration in monkeys with pneumonia (represented by M4 and M6, Fig. 2G). Using necropsy specimens, viral RNA copies were detected in the M0 monkey at 5 dpi and the M1 monkey at 7 dpi in the nose (10^6^–10^8^ copies/mL), pharynx (10^4^–10^6^ copies/mL), lung (10^3^–10^7^ copies/mL), and gut (10^4^–10^6^ copies/mL) (Fig. 3A, left panel). Hematoxylin and eosin (H&E) staining revealed a mild-to-moderate interstitial pneumonia characterized by widened alveolar septa, increased alveolar macrophages and lymphocytes in the alveolar interstitium, and degenerated alveolar epithelia; moreover, infiltrated inflammatory cells were detected in the lungs of monkeys with primary infection. Collagen fiber could also be observed in the thickened alveolar interstitium in M0 and M1 monkeys using Modified Masson’s Trichrome stain at 5 or 7 dpi (Fig. 3B). Furthermore, the mucous membranes of the trachea, tonsils, pulmonary lymph nodes, jejunum, and colon of the M0 and M1 monkeys exhibited inflammatory cell infiltrations (fig. S1, left panel), as well as infiltration with abundant CD4^+^ T cells, CD8^+^ T cells, B cells, macrophages, and plasma cells in lungs, as assessed using immunohistochemistry (IHC) (fig. S2). Viral-infected cells were mainly found in alveolar epithelia and macrophages by IHC on sequential sections (Fig. 3C), as well as in the mucous membranes of the trachea, tonsils, pulmonary lymph nodes, jejunum, and colon (fig. S1), confirming that SARS-CoV-2 caused COVID-19 in rhesus monkeys. Collectively, these data demonstrated that all seven monkeys were successfully infected with SARS-CoV-2 and that the pathogenicity in monkeys is similar to that reported in recent studies (*9*–*14*).

**Fig. 1:**
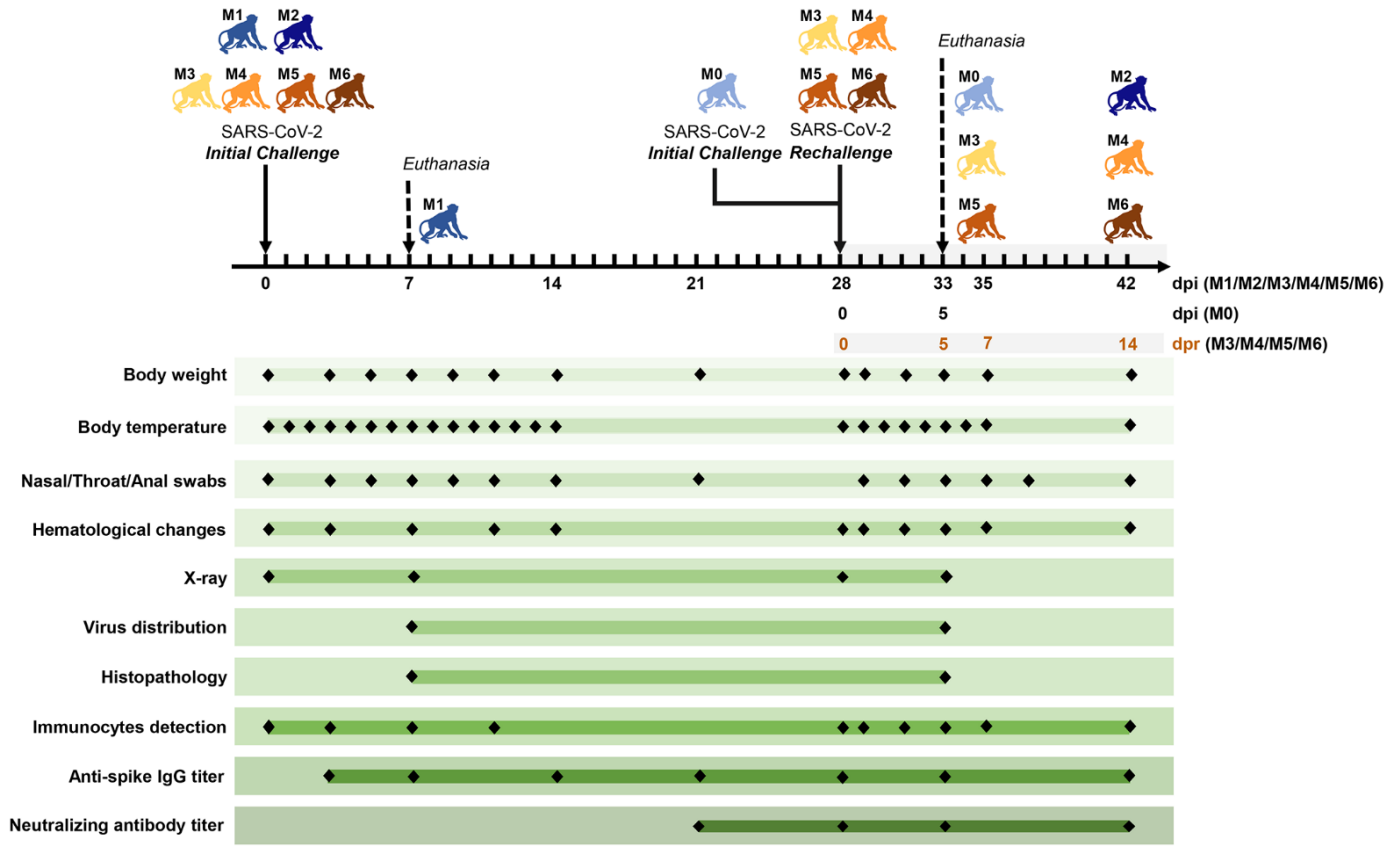
Experimental design and sample collection. Seven adult Chinese-origin rhesus macaques (M0 to M6) were enrolled in the current study. At the outset of this experiment, six monkeys (M1 to M6) were challenged intratracheally with SARS-CoV-2 at 1 × 10^6^ TCID_50_. After all the experimentally-infected monkeys had recovered from the primary infection, four infected monkeys (M3 to M6) were intratracheally rechallenged at 28 days post initial challenge (dpi) with the same dose of the SARS-CoV-2 strain, to ascertain the possibility of reinfection. In addition, an uninfected monkey (M0) was also treated with SARS-CoV-2 as the model control of the second challenge, and a previously infected monkey (M2) was untreated in the rechallenge experiment and was continuously monitored as the control animal. To compare the virus distribution and histopathological changes between the initially infected monkeys and the reinfected monkeys, two monkeys per group (M0 and M1 in the initial infection group, M3 and M5 in the reinfection group) were euthanized and necropsied at 5 dpi (M0), 7 dpi (M1) and 5 days post rechallenge (dpr) (M3 and M5), respectively. Body weight, body temperature, nasal/throat/anal swabs, hematological changes, immunocytes, and specific antibodies were measured over the short-term observation period. Two measurements of virus distribution and histopathology (H&E/IHC staining) were carried out at 5 dpi (M0), 7 dpi (M1), and 5 dpr (M3 and M5). Chest X-ray and neutralizing antibody titers against SARS-CoV-2 were examined at the indicated time points.

**Fig. 2:**
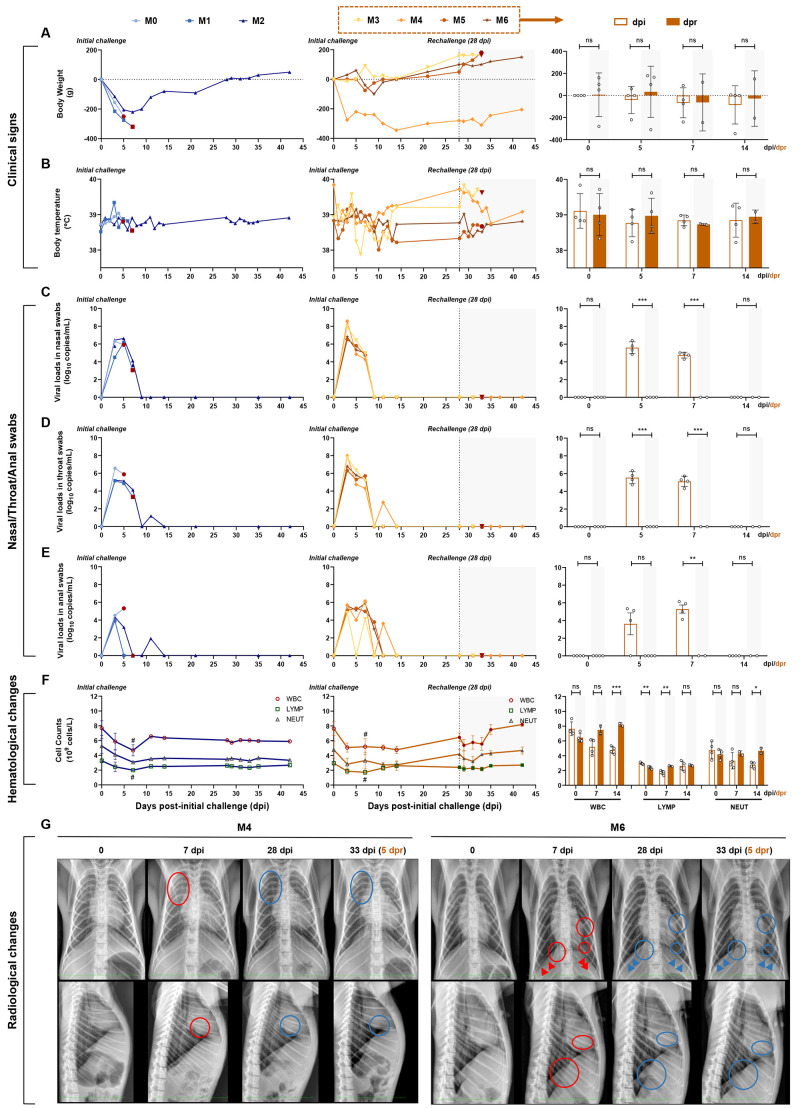
Longitudinal tracking of clinical signs, viral replication, hematological changes, and radiological changes. (**A** and **B**) Clinical signs in each monkey. Monkeys were examined daily for changes in body weight and rectal temperature over the observation period after the initial infection, followed by virus rechallenge. The changes in weight are expressed as body weight loss prior to primary infection. (**C**, **D**, and **E**) Detection of viral RNA in nasal, throat, and anal swabs. The SARS-CoV-2 RNA was detected by qRT–PCR in the swabs from seven monkeys at the indicated time points. (**F**) Hematological changes, including WBC, LYMP, and NEUT counts in the peripheral blood, were monitored. (**G**) Chest X-rays of animals at 0, 7, 28, and 33 dpi (5 dpr) were examined and the representative images of M4 and M6 are shown (red circles, areas of interstitial infiltration and exudative lesion; red arrows, obscured diaphragmatic surface; blue circles and arrows, areas that have recovered from pneumonia). Four monkeys (M3 to M6) were rechallenged at 28 dpi (dotted line and shaded areas), and the results of the initial infection and rechallenge were compared in bar graphs. The bars represent the average of four rechallenged animals at the indicated time points. The viral RNA in nasal, throat, and anal swabs of rechallenged animals were significantly lower than those of the initial infection, while significant hematological changes were observed between the primary and second challenges (unpaired *t*-test, dpi vs. dpr, *P < 0.05; **P < 0.01; ***P < 0.001; ^#^*P < 0.05* 0 dpi vs. 7 dpi).

**Fig. 3:**
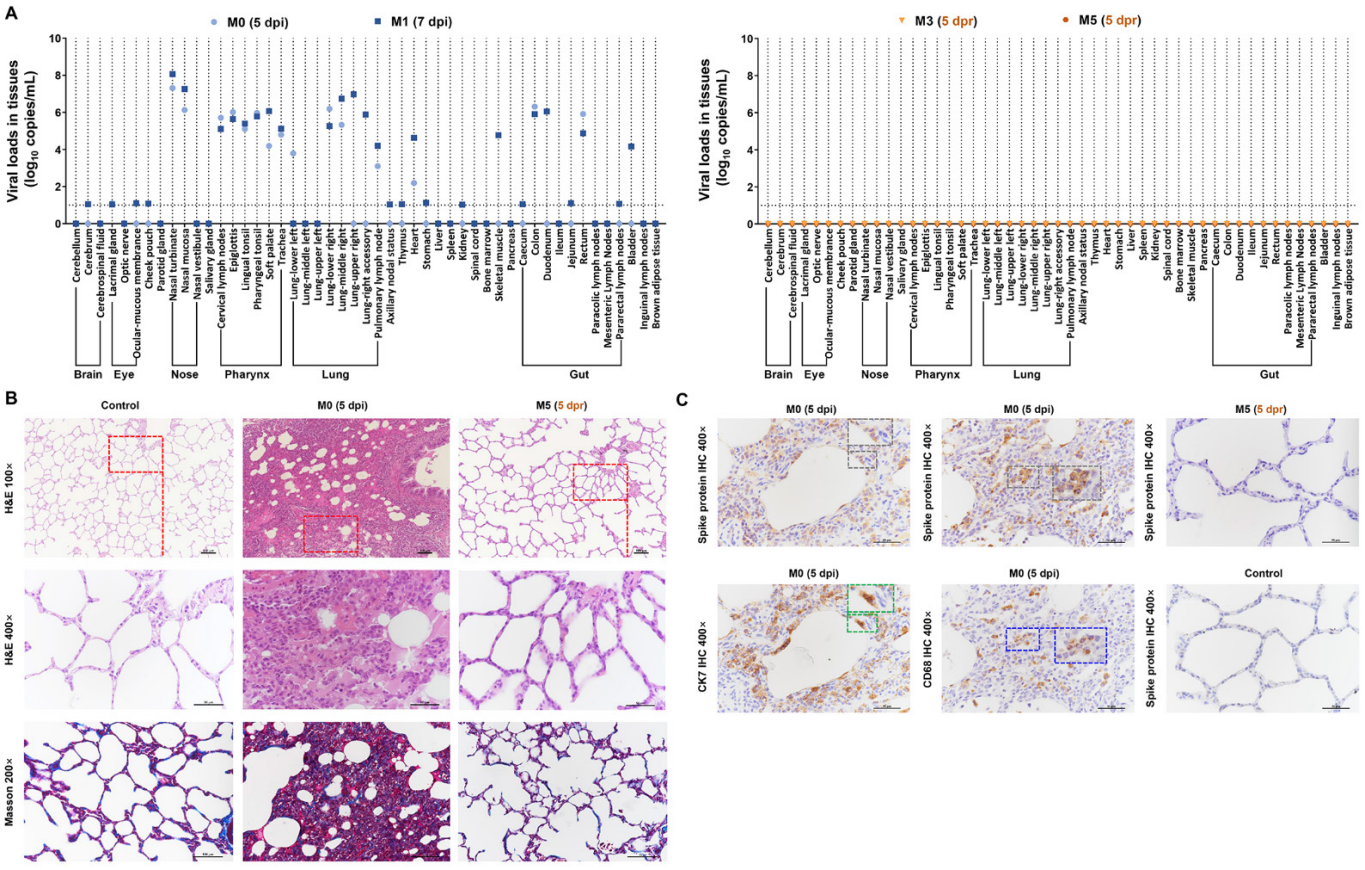
Comparison of virus distribution and pathological changes between the primary challenge and rechallenge stages. (**A**) Detection of viral RNA in the indicated organs (brain, eye, nose, pharynx, lung, and gut. Compared with M0 and M1 (at 5 or 7 dpi; primary infection stage), viral replication tested negative in the indicated tissues from M3 and M5 (at 5 dpr; virus rechallenge stage). Using a viral load > 10 log_10_ copies/mL as the threshold of positivity-tissue-based PCR, tissues from 49 anatomical parts were detected for qualifying virus-infected positivity. Fourteen tissues from the respiratory tract, gut, and heart exhibited SARS-CoV-2-positive cells in both M0 and M1. SARS-CoV-2-positive cells were only observed in the left lower lung from M0 or in the right upper lung, upper accessory lung, skeletal muscle, duodenum, and bladder from M1. The remaining tissues from 29 anatomical parts did not show SARS-CoV-2-positive cells, indicating that these tissues were intact from viral invasion. (**B**) In M0 (5 dpi), an interstitial lesion including remarkedly widened alveolar septa and massive infiltrated inflammatory cells was observed using H&E staining. A mild fibrosis was clearly detected within widened alveolar septa using Masson staining. IHC against the spike protein of SARS-CoV-2 (7D2, grey frame), macrophages (CD68, blue frame), or alveolar epithelial cells (CK7, green frame) are visualized in parallel in Fig. 3**C**. The spike-positive cells overlapped with either alveolar epithelial cells or macrophages showing diffused interstitial pneumonia affected by SARS-CoV-2 invasion. In M5 (5 dpr), no remarked pathological changes and virus distribution were detected via H&E staining, Masson staining, or IHC, indicating that the interstitial lesions had completely recovered from the SARS-CoV-2 primary infection and were intact to reinfection. The red rectangles indicate the areas of magnification. Black scale bar at 100× or 200× = 100 μm. Black scale bar at 400× = 50 μm. Data are representative of three independent experiments.

By 15 dpi, the body weight of infected monkeys (M2 to M6) had gradually increased into the normal range (4/5, the exception of M4, Fig. 2A), and their rectal temperature was maintained within the normal range (Fig. 2B). Moreover, the viral loads were negative in all nasopharyngeal and anal swabs (5/5, Fig. 2, C to E). As shown in Fig. 2F, the hematological changes remained relatively stable within the normal range. Chest X-rays returned to normal levels at 28 dpi (represented by M4 and M6, Fig. 2G). These traits were similar to the hospital discharge criteria used for patients with COVID-19, including absence of clinical symptoms, radiological abnormalities and twice-negative RT–PCR results (*15*). Taken together, our results suggest that monkeys that underwent initial SARS-CoV-2 infection required about 2 weeks to transition into the recovery stage (*10*, *16*).

At 28 dpi, four monkeys (M3 to M6) that underwent primary infection and recovery were rechallenged intratracheally with the same dose of an identical SARS-CoV-2 strain. The clinical tracking of the reinfection included examination of weight loss (Fig. 2A) and rectal temperature (Fig. 2B). Interestingly, the rechallenged monkeys exhibited a transient increase in temperature, which was not observed during the primary infection. Viral loads remained negative over a 2-week intensive detection of the virus in nasopharyngeal and anal swabs after rechallenge with SARS-CoV-2 (Fig. 2, C to E). Peripheral blood measurements revealed no significant fluctuation during the rechallenge stage (Fig. 2F). Moreover, we did not detect abnormalities by X-ray in the M4 and M6 monkeys at 33 dpi (5 dpr, Fig. 2G). In necropsy specimens of the lungs and extrapulmonary tissues of rechallenged monkeys (M3 and M5 at 5 dpr), we found no detectable viral RNA (Fig. 3A, right panel), no significant pathological lesions (Fig. 3B, right panel of fig. S1), no viral-infected cells (Fig. 3C, right panel of fig. S1), and no immune cell infiltration (fig. S2). Therefore, the rhesus monkeys that initially developed primary SARS-CoV-2 infection did not appear re-infected with the identical SARS-CoV-2 strain during their early recovery stage.

To interpret the challenge–rechallenge disparity, we performed a comparison of the clinical, pathological, viral and immunological traits that comprehensively reflected the virus–host interaction between the primary-challenge stage and the rechallenge stage in four monkeys (M3 to M6). First, the viral loads in nasopharyngeal and anal swabs were much higher at 5 or 7 dpi than they were at 5 or 7 dpr (right side of Fig. 2, C to E). An increased WBC and neutrophils were observed at 14 dpr compared with 14 dpi (Fig. 2F, right side). Second, T and B cells from peripheral blood, including CD4^+^ T subsets [naïve CD4^+^ T cells (CD4^+^ T naïve), central memory CD4^+^ T cells (CD4^+^ Tcm), and effective memory CD4^+^ T cells (CD4^+^ Tem)], CD8^+^ T subsets (CD8^+^ T naïve, CD8^+^ Tcm, and CD8^+^ Tem), memory B cells, and plasma cells were relatively stable during the challenge–rechallenge infectious stage. However, an increased percentage of activated CD8^+^ T cells from peripheral blood was observed at 14 dpi, which was also found at 0 dpr compared with 0 dpi (Fig. 4A, right side). Regarding the immune responses from lymph nodes, an increased percentage of CD4^+^ Tcm cells and decreased percentage of naive CD4^+^ T cells and memory B cells from lymph nodes were observed at 5 dpr compared with 5 dpi (Fig. 4B). Third, the specific antibody levels against the SARS-CoV-2 spike increased gradually, leading to a significantly higher titer at 21 dpi than at 3 dpi and 42 dpi (14 dpr) compared with 28 dpi or 0 dpr (Fig. 4C). Moreover, the specific antibody titers were much higher at 14 dpr compared to 14 dpi (Fig. 4C, right panel). As shown in Fig. 4D, the average titers of neutralizing antibodies exhibited a linear enhancement at the time of rechallenge (28 dpi; range, 1:8-1:20 in all monkeys). We observed an enhanced activation of CD8^+^ T cells from peripheral blood, and changes in CD4^+^ Tcm cells and memory B cells from lymph nodes. It appeared an increased number of neutralizing antibodies against SARS-CoV-2 were induced by cellular or humoral immunity facilitated by the primary infection, which might have protected the same nonhuman primates against reinfection in the short term. However, factors that are directly correlated to protection are yet to be fully elucidated. Further studies of passive transfer of convalescent sera from this model to a naïve macaque, or CD8^+^ T cell depletion in the recovered monkeys prior to rechallenge, would be required to define the mechanisms underlying the pathogenicity of SARS-CoV-2.

**Fig. 4:**
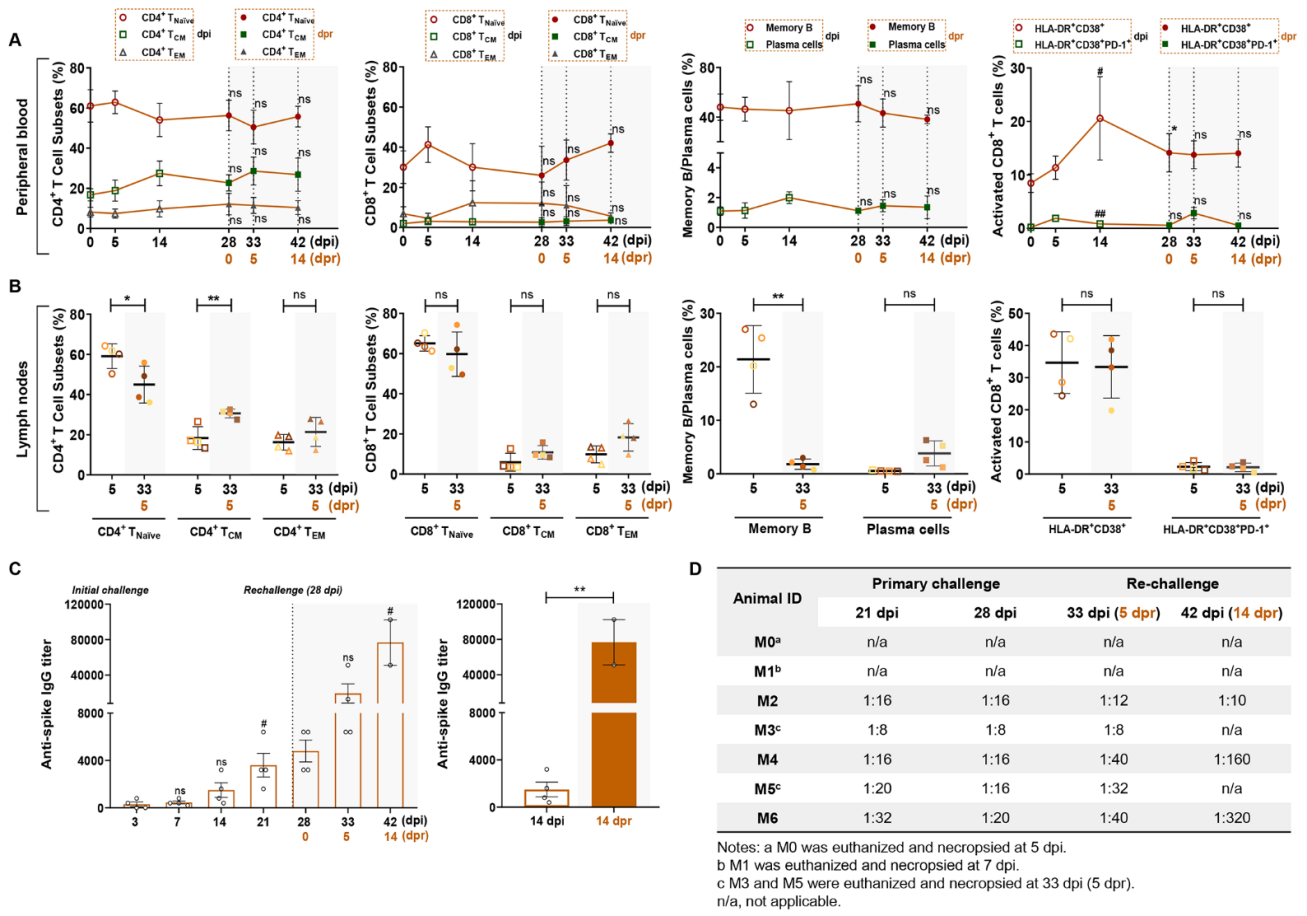
Comparison of cellular and humoral immunity between primary challenge and rechallenge stages in macaques. Four macaques (M3–M6) were rechallenged at 28 dpi (dotted line and shaded areas), and the results of the initial infection and rechallenge were compared at the same time points after the challenge and after the rechallenge. (**A**) Percentages of memory CD4^+^/CD8^+^ T cell subsets, memory B cells, plasma cells, or activated CD8^+^ T cells from peripheral blood for the challenge–rechallenge experiments. Compared with 0, 5 or 14 dpi, there was no significant differences on the percentage of naive CD4^+^/CD8^+^ T cells (CD4^+^/CD8^+^ Tnaïve, CD3^+^ CD4^+^/CD8^+^ CCR7^+^ CD45RA^+^), central memory CD4^+^/CD8^+^ T cells (CD4^+^/CD8^+^ Tcm, CD3^+^ CD4^+^/CD8^+^ CCR7^+^ CD45RA^−^), effective memory T cells (CD4^+^/CD8^+^ Tem, CD3^+^ CD4^+^/CD8^+^ CCR7^-^ CD45RA^−^), memory B cells (CD3^−^ CD20^+^ CD27^+^) and plasma cells (CD3^−^ CD20^+^ CD43^+^) from peripheral blood at 0, 5, 14 dpr (unpaired *t*-test, ns, *P* > 0.05). The activation of CD8^+^ T cells (CD8^+^ CD38^+^ HLA-DR^+^ or CD8^+^ CD38^+^ HLA-DR^+^ PD-1^+^) at 14 dpi were increased compared with the baseline (unpaired *t*-test, ^#^*P < 0.05, ^#^*^#^*P < 0.01*), and elevated levels of CD8^+^ CD38^+^ HLA-DR^+^ T cells were also observed at 28 dpi (unpaired *t*-test, 0 dpi vs 0 dpr, * *P < 0.05*). (**B**) Percentages of memory CD4^+^/CD8^+^ T cell subsets, memory B cells, plasma cells or activated CD8^+^ T cells from lymph nodes between 5 dpi and 5 dpr. An increased percentage of CD4^+^ Tcm cells and decreased percentage of CD4^+^ Tnaïve cells and memory B cells from lymph nodes were found in the dot plots (unpaired *t*-test, *P < 0.05, **P < 0.01). (**C**) Levels of specific IgG against the spike protein of SARS-CoV-2 in four rechallenged monkeys. The levels of anti-viral antigen-specific IgG from each monkey were detected at 3, 7, 14, 21, 28, 33, and 42 dpi. Significantly increased levels of IgG were observed between the primary and second challenges (unpaired *t*-test, **P < 0.01, 14 dpi vs. 14 dpr; ^#^ *P < 0.05,* 3 dpi vs. 21 dpi, 28 dpi vs. 42 dpi). (**D**) Neutralizing antibody titers for protection of SARS-CoV-2-infected monkeys against reinfection.

In the present challenge–rechallenge infection of rhesus monkeys with SARS-CoV-2, observations and detections were carried out within the relative short time window in which neutralizing antibodies plateaued after the primary infection. A longer interval (longer than 6 months) between the primary challenge and the rechallenge is needed to track longitudinally the host–virus interaction and elucidate the protective mechanism against SARS-CoV-2 in primates. Moreover, all infected monkeys exhibited relative mild-to-moderate pneumonia, which is similar to the mild or common clinical characteristics of COVID-19 in infected human individuals; however, a COVID-19 monkey or transgenic mouse model with severe clinical symptoms or lethality should be explored according to increasing challenge dose, exposed tissues, or other treatments. Although we observed macrophages in association with SARS-CoV-2 infection, rapid rechallenge in the absence of potent serology relieved some level of concern regarding the occurrence of ADE. In addition, mucosal immunity, which is triggered by primary infection and includes both the respiratory and intestinal mucosa and local lymph nodes, might contribute substantially to the viral rechallenge response. Thus, future studies are required to examine experimentally the mucosal antibody responses, such as bronchoalveolar lavage IgG levels, serum IgA or IgM levels.

Taken together, our results suggest that rhesus macaques that have undergone an initial infection with SARS-CoV-2 mount protection against rechallenge during the early recovery days. However, it remains necessary to elucidate the protective mechanism against SARS-CoV-2 regarding neutralizing antibodies or other immunological roles. This short-term infection rechallenge macaque model provides insightful information for vaccination research, therapy of convalescent sera, and prognosis of COVID-19.

## Supplementary Materials

science.sciencemag.org/cgi/content/full/science.abc5343/DC1 Materials and Methods Figs. S1 and S2 References (*17*)
